# Spotlight on early-career researchers: an interview with Yi Zhu

**DOI:** 10.1038/s42003-018-0211-7

**Published:** 2018-12-12

**Keywords:** Careers, Lab life, Obesity

## Abstract

Yi Zhu is an Instructor at the University of Texas Southwestern Medical Center and an NIH K99/R00 career development award recipient. In this interview—part of our series on early-career researchers—he tells us about his work studying metabolic diseases and how he overcame doubts about his career in research by finding out the grass is not always greener on the other side. Finally, Dr. Zhu shares some great advice about finding balance in life, which is important for early-career researchers and anyone else pursuing a busy and challenging career.

**Figure 1:**
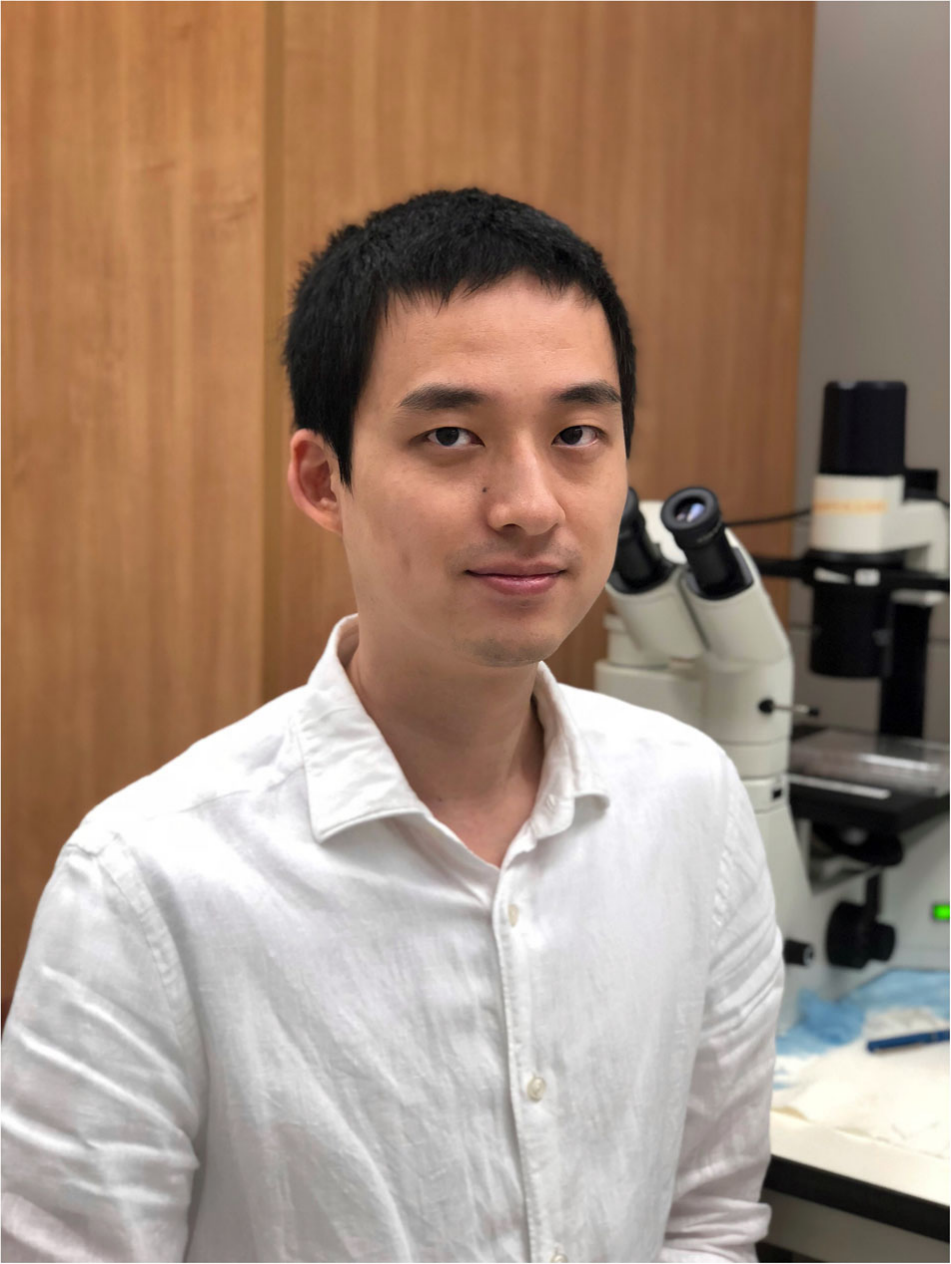
Image credit: Yi Zhu

Can you tell us about your research interests?

I am interested in understanding the pathophysiology of obesity and metabolic diseases and finding new ways to treat them. I am currently focusing on a very specific area, with a very ambitious aim: understanding how adipocytes communicate with each other using gap junctions and how this affects adipose tissue remolding and its communication with other organs.

What has your journey been to this point?

My journey to this point has been propelled by a combination of passion for science and personal decisions made at a few important crossroads in my life. I have always been curious about how nature works, especially the human biological system, so I studied biology in college in China. I then decided to come to the U.S. for a Ph.D. because of the caliber of training available here.

In graduate school, I became interested in metabolism and chose my Ph.D. mentor’s lab largely based on my interactions with him. I held the belief that good mentors nurture and challenge trainees to achieve their full potential. Graduate school does not just involve carrying out experiments and publishing—having a mentor who is a role model and is willing to mentor others is also very important. A good mentor will support one’s development and help the mentee persevere through the challenges of graduate school.

A science career is not always filled with the glory of doing cutting-edge research, achieving breakthroughs, and publishing in impressive journals. One will inevitably feel a sense that progress has slowed at times and will experience occasional setbacks. Three or four years into graduate school, I experienced doubts about whether I had chosen the right career, and I began looking for alternatives. I even took action to prepare for an alternative career, completing chartered financial analyst (CFA) tests during a 3-year period spanning my last 2 years of graduate school and the first year of my postdoctoral training. After those attempts, I realized that the grass often just appears greener in other people’s yards—challenges and frustrations are a natural part of every career path—they do not signal that we have made a poor choice. I then felt more at peace with my career choice and grew more devoted to a career in science.

All the struggles in life prepare us for the opportunities, I realized. When Eli Lilly offered a fellowship that would allow me to work in drug discovery, I grabbed it and worked for them for 3 years. Then I came back to academia because of the freedom it provides in one’s research. I am now looking to transition into becoming an independent investigator with a K99/R00 career development grant from the NIDDK. I feel that I am at an exciting juncture in that I will finally have my own lab soon.

What are your predictions for your field in the near future?

It is very difficult to make predictions within the sciences. This uncertainty is the beauty of science and serves as adrenaline for curious people. I feel the NIH demonstrates a similar philosophy in sponsoring as many small projects as they can.

However, in terms of drug discovery for metabolic diseases, I want to make a few bold and probably biased predictions:

First, the leveraging and modification of natural hormones remains a powerful way to modulate metabolism, control body weight, and correct metabolic abnormities. Great progress has been made in treating diabetes with GLP1 and creating artificial dual agonists or tritagonists that simultaneously target GLP, GIP, and glucagon receptors. Why have these strategies shown stronger efficacy than simply combining two or three hormones? Is the stoichiometry important for those molecules? Glucagon is a hormone that increases hepatic gluconeogenesis, but the incorporation of glucagon component significantly enhances the weight-loss effect. I believe the current trends in the field signal that this is the perfect time to revisit the function of glucagon and other canonical hormones, which can now be studied more comprehensively with the presence of more advanced technology.

Another major development in the field is the rethinking of drug discovery for heart failure, especially conditions with reduced or preserved fractional shortening. The hospital mortality for heart attacks has been significantly reduced over the past century, largely due to many classes of drugs and care procedures that have been introduced for heart failure. However, essentially no drug directly modulates the heart’s metabolism. The recent success in clinical trials of diabetes drugs such as the SGLT2 inhibitor and GLP1 in reducing cardiovascular deaths prompts interest in understanding heart failure in a setting of metabolic intervention. Whether the SGLT2 inhibitor has a direct effect on cardiac metabolism or exerts its cardiac protection via hemodynamic regulation, or both, is worth exploring. Studies should examine whether targeting cardiac metabolism or insulin-signaling pathways—a master regulator of cardiac metabolism—would be the most viable new approach for developing drugs for heart failure.

Last, nonalcoholic hepatosteatosis is an exciting emerging area of study on metabolic diseases in the liver organ. Many clinical trials are testing the ideas and approaches that various biotech and pharmaceutical companies have taken. Of all the potential strategies for combatting NASH, metabolism—especially lipid metabolism—is one of the most important areas that should be explored in designing drugs to treat this disease.

Can you speak of any challenges that you have overcome?

For me, the great challenge is how to stay focused on pursuing a science career. As a human being, I have experienced distractions within my personal life as well as weariness with my studies. I still experience them from time to time, but I can handle them better now. Once, I listened to an interview in which a war veteran—supposedly a fearless fighter for the country—said he was not fearless; he just had a good relationship with the fear. I believe the same idea applies for all of us. We must acknowledge that we have those feelings and find inner peace in knowing that they are a core part of human nature. Then we can find ways to deal with them. The true challenge is to do a little bit better each day.

Regarding specific issues that may affect early-career researchers, I feel the two most prominent challenges are time management and personnel management. People advocate for living a balanced “life”, but no one ever says to live a balanced “day” or “week”. We have to accept that our lives can lose balance at a certain stage, and sometimes we have to make certain sacrifices. Thus, we must know what we desire and how intensely we desire it. Even with a mindset of acceptance that we will need to work hard to succeed in this field, we still cannot stretch a day beyond 24 h. We must therefore learn to say no to some tasks that act as distractions from more critical work, in order to prioritize and improve our efficiency.

In regard to personnel management, learning to recruit and manage a lab is just the beginning. Managing relations with one’s family, senior staff, peers, and subordinates should hold priority as well. I believe communication is the most important key to success in this area. Clarifying one’s own position, understanding the position of others, finding common ground, and resolving the differences sometimes even generates new options that neither party had thought of, achieving a win-win scenario.

What advice would you give to your younger self?

I would not have a lot of advice for my younger self, as I do not regret much and have made peace with my past. There is a meaning to all the mistakes or missteps we make if we learn from them. They become tuition rather than a loss. When I used to look at success stories, I would attribute them to a few qualities in others and hope to replicate those stories by achieving those few arbitrarily selected qualities. In one sentence, I would want to tell my younger self that success can rarely be replicated, and failure provides more insight than a success story.

One thing that many different versions of “Chicken Soup for the Soul” about success get right is the importance of working hard. The process of producing research is less glamorous than what a paper can show, usually requiring thousands of hours of laborious work. Succeeding in science takes a combination of endurance, persistence, and bravery. There is no shortcut to becoming successful in science; one can simply avoid some detours by learning from past mistakes or others’ failures.

Lastly, all those who enter the field should remember that working in scientific discovery is not just a job; it is a noble devotion to creating a better world.

What lessons did you learn from your research, which you hope to apply to your everyday life?

I possess instincts shaped by my past experiences. Thus, I have usually followed my feelings in making decisions in my life. However, feeling can be biased or misleading; I therefore hope I can become more analytical in other aspects of life outside of research. My research has also shown me the need to maintain a singular focus on a task rather than multitasking. In the lab, when time was tight, I used to try to take on several things at the same time, but one or more of the experiments usually went awry. Our brains may be able to take on different tasks in parallel, but only one thing can have its unshareable attention. I now strive to apply this lesson to my everyday life, focusing on the most important thing first and fully devoting my focus to everything that I do.

*This interview was conducted by Associate Editor Jung-Eun Lee*.

